# Binge-Eating Precursors in Children and Adolescents: Neurodevelopment, and the Potential Contribution of Ultra-Processed Foods

**DOI:** 10.3390/nu15132994

**Published:** 2023-06-30

**Authors:** Esther Via, Oren Contreras-Rodríguez

**Affiliations:** 1 Child and Adolescent Mental Health Research Group, Institut de Recerca Sant Joan de Déu, Santa Rosa 39-57, 08950 Esplugues de Llobregat, Spain; 2 Department of Child and Adolescent Mental Health, Hospital Sant Joan de Déu, Passeig Sant Joan de Déu, 2, 08950 Esplugues de Llobregat, Spain; 3 Medical Imaging, Girona Biomedical Research Institute (IdIBGi), Parc Hospitalari Martí i Julià-Edifici M2, Salt, 17190 Girona, Spain; 4 Health Institute Carlos III (ISCIII) and CIBERSAM, 28029 Madrid, Spain; 5 Department of Psychiatry and Legal Medicine, Faculty of Medicine, Universitat Autònoma de Barcelona, 08193 Bellaterra, Spain

**Keywords:** binge eating, emotional eating, ultra-processed food, reward, inhibition, MRI, children, adolescents

## Abstract

Binge-eating disorder (BED) is a highly prevalent disorder. Subthreshold BED conditions (sBED) are even more frequent in youth, but their significance regarding BED etiology and long-term prognosis is unclear. A better understanding of brain findings associated with BED and sBED, in the context of critical periods for neurodevelopment, is relevant to answer such questions. The present narrative review starts from the knowledge of the development of emotional self-regulation in youth, and the brain circuits supporting emotion-regulation and eating behaviour. Next, neuroimaging studies with sBED and BED samples will be reviewed, and their brain-circuitry overlap will be examined. Deficits in inhibition control systems are observed to precede, and hyperactivity of reward regions to characterize, sBED, with overlapping findings in BED. The imbalance between reward/inhibition systems, and the implication of interoception/homeostatic processing brain systems should be further examined. Recent knowledge of the potential impact that the high consumption of ultra-processed foods in paediatric samples may have on these sBED/BED-associated brain systems is then discussed. There is a need to identify, early on, those sBED individuals at risk of developing BED at neurodevelopmental stages when there is a great possibility of prevention. However, more neuroimaging studies with sBED/BED pediatric samples are needed.

## 1. Introduction

Binge-eating disorder (BED) has been a diagnosis on its own only since the last edition of the Diagnostic and Statistical Manual of Mental Disorders (DSM-5) [1] in 2013. However, data on prevalence already suggest it is the most-prevalent eating disorder worldwide [2]. The first peak of prevalence occurs in adolescence [3], at 1–3% in children and adolescents [3], and 3% for subclinical presentations [4]. BED prevalence rises to 37% in populations of adolescents with obesity [5,6]. Despite the high prevalence, full comprehension of the diagnosis and its pathophysiological mechanisms is still in its infancy, particularly regarding youths, who frequently have a different clinical presentation compared to adults [7].

BED involves at-least-weekly episodes of binge eating, which imply eating a large amount of food in a discrete time period and with a sense of lack of control over it [1]. It is associated with physical and psychiatric comorbidities and a high impact on quality of life and disability-adjusted life-years (DALYs) [2,8]. Regarding physical comorbidity, BED is most frequently associated with obesity, which confers a risk of other medical conditions such as metabolic and cardiovascular diseases, leading causes of death worldwide [9]. Psychiatric comorbidities are the norm more than the exception, representing more than 60% lifetime prevalence in subjects with BED, mostly anxiety and mood disorders [10,11]. While BED is a recognized disorder, other related presentations have attracted interest in the literature. These presentations are either subclinical or precursor forms of BED (from now on, sBED), which are defined by different but clinically overlapping terminology throughout the scientific literature. Such constructs are not validated, are mostly descriptive, and most do not use operationalized criteria. In some cases, but not all, individuals with these conditions might be diagnosed with specified or unspecified DSM-5 eating disorder categories (“other specified feeding or eating disorder”, OSFED, or “eating disorder not otherwise specified”, EDNOS) [1]. Some examples are *disordered eating* [12], *dysregulated eating behaviour* [13], *uncontrolled eating* [14,15], *emotional eating* [16], *overeating disorder* [15], *disinhibited eating* [17], *LOC eating* (loss of control overeating), the latter being the sole condition with operationalized criteria [5,18,19]. Another closely related concept is *food addiction*, a construct under considerable debate for the last 10 years that entails compulsive and dysregulated intake of high-calorie foods [20,21,22,23]. Food addiction is a transdiagnostic construct; it is present in non-clinical and clinical samples. For example, it is found in high percentages in clinical samples of eating disorders (between 41.5% and 72.2% in BED) [24].

In comparison with adults, incomplete sBED forms are more frequent in youths, which is partially explained by the developmental differences between the two groups [7]. For example, binges in children generally entail lower energy intake, and unadjusted behaviour is likely limited by the environment (i.e., limited access to food) [7]. Considering this, adapted criteria have been proposed to detect BED in youths, although this is not a DSM-5 validated diagnosis and it is not widely used in the literature (see refs. [25,26,27,28]). BED diagnoses are frequently preceded by sBED in paediatric populations, with symptoms such as *LOC eating*, eating in response to emotions, eating without hunger, and overeating being associated with a higher risk for later BED development in different studies [18,19,29,30]. Of note, the prevalence of sBED in children is stable across childhood [31] and the presence of sBED in childhood is associated with a higher risk, compared to adulthood, for more severe binge eating and other eating disorders such as bulimia nervosa [19,32]. The rate of diagnostic conversion from sBED to BED is not clear, and to our knowledge, only one study has evaluated this matter, reporting 28% transition from sBED to BED in a group of adolescents during a 8-year of follow-up [33]. Given that it was a community study, the sample of sBED was very small (*n* = 18).

The most important known underlying factor in BED and sBED is deficiency in emotional regulation strategies [34,35], which refers to the ability to regulate emotional responses and to inhibit impulses for immediate gratification in the service of waiting for larger, delayed rewards [36]. In this scenario, binge-eating episodes are considered maladaptive strategies to cope with negative affections (e.g., sadness, boredom, restlessness) and/or to obtain rewarding experiences [7,37]. Indeed, difficulties in emotional identification and awareness, impulsivity, reward sensitivity, depressive symptoms, low self-esteem, anxiety, ruminative tendencies, and the presence of an attention deficit and hyperactivity disorder (ADHD) have been found to predict sBED or to be associated with binge-eating scores [19,22,35,38,39,40,41,42]. For ADHD, the overlapping of implicated brain circuits in the two pathologies suggests common neurological substrates or pathophysiological processes [15]. Notably, brain circuits underlying emotional regulation processes develop during childhood and adolescence, coinciding with the emergence of sBED. While some research has been conducted on these circuits in the context of sBED/BED, for example, to evaluate prefrontal responses to inhibition-processing demands, or reward processes at the level of the nucleus accumbens—among other regions—our understanding of the relationship between the development of underlying emotion-regulation brain systems in youth and sBED/BED remains limited. In this regard, some studies have suggested that sBED and BED are mediated by premorbid deficits in neural systems regulating emotions which confer a vulnerability for sBED/BED development [19,35,43].

In contrast, other studies have suggested that dietary restraint is involved in the development of BED (restraint model) [44,45,46,47], while others have implied that sBED, and possibly BED, originate in long-term hypercaloric food consumption [35]. The literature supporting the former is rather mixed, and results might depend on the evaluated outcome (i.e., bulimia nervosa, BED or sBED), the sample characteristics (i.e., subjects with obesity vs. community samples), the variables studied (i.e., fasting, low-calory diets, regularity of meals), and the evaluation of other concomitant factors (i.e., dieting due to internal motivations related to body dissatisfaction vs. other reasons for dieting) [44,45,46,47,48,49,50]. Once BED is established, however, regular eating and no skipping of meals are two of the most important factors to overcome the disorder [49]. Regarding the long-term hypercaloric food consumption hypothesis, recent evidence points towards an association between high consumption and exposure to ultra-processed foods and drinks (UPF in advance) and the development of sBED and BED [51,52]. In addition, current evidence links UPF consumption with sBED/BED-associated brain systems (see further). Children and adolescents show high rates of UPF consumption, with estimates indicating that they obtain between 29% and 68% of their total energy intake from UPF consumption, a figure which increased by 5.6% between 1999 and 2018 [53,54]. In Spain, the percentage of UPF among all food purchases almost tripled between 1990 and 2010 (from 11.0% to 31.7%) [55]. Notably, this previous data are concurrent with the alarming rise in obesity prevalence in youth [9,53,56,57].

Given that eating disorders are better understood from a biopsychosocial framework [58], it is important to note that other factors are also important contributors to the development of sBED and BED. The etiology of eating disorders is indeed multifactorial and complex, characterized by a dynamic interplay among biological factors (e.g., inheritable traits, neurodevelopmental influences, neuroendocrinological factors), as well as psychological (e.g., personality traits such as low self-esteem) and environmental factors (e.g., sociocultural expectations) [58,59]. Moreover, all of these factors can both trigger and perpetuate eating disorders [59]. In the context of children and adolescents and sBED/BED, one important factor includes food-learning habits, for example through parental style (extensively studied, as in [60,61,62]); parents might use practices such as restricting food access to the child, pressuring the child to eat, or using food as a reward or in an attempt to regulate negative emotions [62]. Such a relationship, however, is seen to be complex and bilaterally associated with child behaviour [60]. Nevertheless, the contribution of all these variables to sBED/BED are beyond the scope of this review and will not be covered here.

A better comprehension of the link between sBED conditions and BED pathophysiology, both clinically and at the level of the brain, as well as the long-term trajectory of these two forms during vulnerable neurodevelopmental periods, might help to detect and treat those individuals at greater risk for BED [63]. In addition, it is also necessary to review the evidence of the association between the consumption of UPF, BED, and sBED forms, in order to identify and examine new factors that may contribute to increasing the clinical and subclinical presentation of this eating disorder.

### Review Scope

In the present narrative review, we aim to provide a clear picture of the convergence between brain alterations in sBED in children and adolescence, and those under full BED forms. To that end, we will first provide a brief review of the brain circuit’s underlying eating behaviour (Section 2), as well as of the neurodevelopment of emotional self-regulation processes (Section 3). In Section 4, we will synthetize the neuroimaging studies conducted on sBED and BED, and draw conclusions regarding the convergence and divergence of the findings, if possible. The preliminary evidence linking the high consumption of UPF, sBED, and BED is reviewed in Section 5. In Section 6, we conclude by suggesting lines of research for future studies. Despite not conducting a systematic review (while others exist, such as [13]), a selection of reviewed articles has been conducted using a systematic search on three different databases (Pubmed, Scopus, WoS), specifically looking for magnetic resonance studies in children and adolescents and on binge eating or subtheshold conditions such as emotional eating or food addiction. We apologize in advance to our colleagues whose work has been omitted unintentionally or due to space constraints.

## 2. Eating Brain Circuitry

Eating behaviour is driven by several psychobiological factors that include homeostatic factors, the coding of rewarding properties of food, and other individual psychosocial factors associated with eating [64]. Food is processed through ascending sensory pathways that bring information about the properties of food (smell, taste, texture) to the brain. For example, gustatory information is processed by the cranial nerves, the nucleus of the solitary tract, and the gustatory thalamus [65]. Information from different sensory channels is then largely integrated to the frontal opercula and insular cortex [65,66,67,68,69]. Next, the orbitofrontal region (its caudolateral parts considered the secondary cortical taste) assigns reinforcement values to food [70,71]. Other limbic and cognitive neural systems involved in eating behaviour modulate this primary response, for example, in the anticipation of the food stimulus, in the hedonic or emotional response, and in approaching behaviour related to food stimuli [72]. These regions might be conceptualized as a ventral system (emotional, excitatory) and a dorsal system (cognitive, inhibitory). This model has been used to explain other eating disorders, such as anorexia nervosa [73].

The ventral system is composed of the striato-limbic and ventral parts of the prefrontal cortex, and it sends information to more dorsal prefrontal pathways (bottom-up). It is involved in the monitorization of internal and external responses, such as the identification of emotional significance, encoding the value for a choice, and in hedonically motivated eating behaviours, including craving [72,74,75,76,77]. For example, the orbitofrontal cortex and the striatum (dorsal- and ventral-encompassing the nucleus accumbens, the hub area of the reward system) are involved in both the anticipation and the consummatory food reward [78,79,80,81]. Within this circuit, the insular cortex is a multimodal region that integrates primary sensory and interoceptive information with emotional, cognitive, and motivational signals in a posterior-to-anterior anatomical progression [69,82,83]. Together with the thalamus, it encodes the organoleptic properties of food and food energy, and regulates energy balance, feeding, and satiety [67,84]. As an example, the response of the insular cortex is associated with gut hormone responses and peripheral blood glucose levels [84,85]. Thus, the insular cortex plays an important role in homeostasis and interoceptive processes involved in eating behaviour [86,87]. In addition, anterior parts of the insula have been consistently involved in craving, including food craving [88,89,90].

The dorsal system (top-down), in turn, includes prefrontal regions, and it exerts control over subcortical striato-limbic structures [73,91]. This neural system is in charge of executive functions, such as planning and organization for problem solving, which are crucial to direct behaviours toward objectives, and to inhibit behaviours [73]. For instance, it is critically involved in the decision to eat or to inhibit the desire (or craving) to eat [92]. Under physiological conditions, the balance functioning between these ventral and dorsal neural systems, and their interaction with homeostatic regulatory circuits in the hypothalamus, will finally determine food intake (for a review, see [93,94]). Alterations within the ventral and dorsal systems are thought to characterize sBED/BED [95]. The next section will provide a review of our knowledge regarding the standard neurodevelopment of brain systems involved in emotional self-regulation and the potential implication for the risk of developing BED.

## 3. Neurodevelopment of Emotional Self-Regulation

As mentioned, deficits in emotional regulation processes crucially underlie sBED and BED [34,35]. Adaptive self-emotional regulation is achieved over life by learning processes, with cognitive reappraisal being the most studied. Cognitive reappraisal entails deliberately altering the self-relevant meaning (an appraisal) of an emotion-inducing stimulus to change its emotional impact [96]. Its frequent use is linked to an improved control of emotions, cognitive performance, and interpersonal functioning [97]. Children can engage in cognitive reappraisal with adult guidance between the ages of three and five but it is not until middle childhood, around 6–7 years of age, that they are able to independently reappraise emotional stimuli if instructed to do so (for a review see [98]).

The effective employment of cognitive reappraisal is dependent on underlying executive functions, such as working memory and attentional shifting [99]. In congruence with the normal development of prefrontal-supporting executive systems, neuropsychological and neuroimaging studies suggest that this strategy is not effective until adolescence [98]. Such studies indicate that from middle childhood into late adolescence, the use of reappraisal effectively downregulates the activity within core limbic brain structures involved in emotion generation (e.g., amygdala) [98] (for a review of the network implicated in cognitive reappraisal, see [100]). Among the diverse prefrontal areas implicated in cognitive reappraisal, the ventrolateral and medial prefrontal cortices have been repeatedly associated with the effectiveness of reappraisal in studies, with samples ranging from middle to young adulthood [101]. These regions are associated with the appropriate inhibition of automatic appraisals and the selection of alternate ones, and with the representation of the reward value of goal-directed behaviours [100]. Children, with a yet immature prefrontal system, are more dependent on external regulation, mostly from parents [102]. Other factors, such as the temperament of the child and biological factors such as functional non-pathological differences in neurotransmitter receptors (serotonin, dopamine) contribute to these processes [103,104,105].

Thus, childhood and adolescence are key periods for learning emotional regulatory strategies, which are supported differentially across ages. While young children depend greatly on the presence of consistent environment control over the predominance of more limbic and reward-based own drivers, middle children and adolescents rely more on their own self-regulatory strategies, supported by their prefrontal cortex systems and built on temperament characteristics and learning experiences [106]. Although the emergence of sBED and BED may be more evident in adolescents, when autonomy is gained [13], altered relationship with food- and brain-based dysfunction might have started earlier, for example, with learning processes fostered by the immediate environment.

## 4. sBED-Related Brain Vulnerability Markers and BED

This section will first review the studies conducted with samples presenting sBED conditions, and then summarize those conducted with subjects with a BED diagnosis across the main clinical domains of dysfunction.

### 4.1. Response and Behavioral Inhibition Deficits

Several studies suggest that primary deficits in inhibitory functions, supported by prefrontal regions, may underlie the deficits in emotional regulation that confer a vulnerability for sBED and BED [19]. In particular, children or adolescents with such deficits might feel overwhelmed and might fail to cope using adaptative responses [19] when exposed to stressful and negativity-inducing situations (e.g., threats and social losses). In such situations, vulnerable subjects may show a rush for eating (usually palatable) food, which avoids facing emotions and initially reduces the negative affect by the obtainment of pleasure [19,107,108,109]. While this model has been more extensively studied in binges occurring in bulimia nervosa [99], there is some limited evidence for sBED and BED.

At the neural level, some studies give support to deficits in behavioural inhibitory functions in sBED. For example, in adolescents with sBED, one study showed evidence of decreased activation of the frontoparietal and temporal regions during inhibitory processing in a functional magnetic resonance (fMRI) study using the go/no-go task [110]. Similarly, in another study, adolescent girls with sBED and obesity failed to engage prefrontal regions (ventromedial and dorsolateral prefrontal cortices) in an emotion regulation task in the context of negative mood induction through a peer-interaction paradigm [111]. In another pilot study, preadolescents with overweight or obesity and sBED were exposed to an intermittent food restriction paradigm during a magnetic resonance session, in which they received different milkshake flavours in a restricted vs. unrestricted manner [112]; sBED subjects, compared to weight- and sex-matched controls, presented increased activity in self-regulatory and attention regions (right prefrontal regions, left cingulate, and left cuneus) during restricted conditions. Hyperactivations were suggested as representing an increased cognitive effort to regulate emotions under such restrictive conditions [112].

Longitudinal studies using community samples might be more informative in disentangling whether these alterations are or are not primary deficits. Most of these studies come from large longitudinal cohorts in healthy children and adolescents, such as the ABCD study (United States population [113]) or the IMAGEN study (European population [114]). For example, a study from the IMAGEN cohort (*n* = 1607) showed that greater responses of the anterior cingulate cortex and medial prefrontal cortex during failed inhibition trials in a stop signal task at 14 years of age were associated with the development of disordered eating behaviours at 16 (self-reported binge eating and purging episodes), compared to healthy controls [107]. The authors suggested that the increased activation may work as an early compensatory mechanism for inhibitory deficits, which could point towards a potential early biomarker of sBED. Importantly, the brain alterations reported by all these studies were not accompanied by deficits in task-behavioural responses, suggesting inhibitory control performance is not necessarily impaired in sBED, and it also indicates that compensatory mechanisms may be effective. Further studies should elucidate whether such prefrontal hyperactivation is or is not a useful biomarker of sBED and/or its transition to BED.

In BED, evidence of poor impulse control or decreased inhibitory control comes mostly from limited examinations of adult samples (see reviews in [43,91,115]). In the systematic review and meta-analysis in obesity of Lavagnino et al. [43], the authors concluded that, while subjects with obesity (adults and youths) showed decreased inhibitory control performance, such performance did not differ between subjects (only adults) with BED and those without BED. In contrast, a decreased neural activation in prefrontal areas during inhibitory tasks characterized adult subjects with BED and obesity compared to subjects with obesity and without BED in two other studies [116,117].

In conclusion, the very limited literature found in children and adolescents gives some support for alterations in brain regions involved in inhibition control as a potential early dysfunction that facilitates sBED. Based on the reviewed studies, the different directions of the findings (i.e., hypo- vs. hyperactivations) might depend on age (i.e., younger ages presenting hyperactivations), the premorbid stage vs. consolidated sBED (i.e., hyperactivations prior to sBED development) or even the nature and potential triggering effects of the tasks used (i.e., hyperactivations during a simulation of intermittent restriction vs. hypoactivation during tasks purely evaluating cold-cognition, such as the go/no-go task). There is a lack of information in BED on youths, and the literature is mixed on adult samples.

### 4.2. Reward-Based Deficits

Other studies have suggested that some youth, considered vulnerable to sBED/BED and to obesity, might present either a hypo- or hyperresponsive reward system, which can drive them towards developing sBED or BED [118,119]. However, according to Stice and Burger [118], there is little support for the reward deficit theory [14], while the hyperresponsiveness hypothesis is nowadays the theory with the largest support. In this respect, some authors have suggested that the impulsivity that characterizes children with sBED may be explained by an increased sensitivity to reward and decreased ability to delay gratification [120]. Subjects with full BED forms clinically present increased preference for immediate (food or other stimuli) reward as opposed to delayed [109], greater food-reward sensitivity, and greater rash-spontaneous behaviour in the context of food [121].

Some studies have reported neural differences in response to reward in samples with sBED conditions. For example, in one study of healthy children, the symptom “eating in the absence of hunger” was positively associated with the activation of the nucleus accumbens [74]. In community-based cohorts, one study of the ABCD project observed that certain structural differences of key reward brain regions (i.e., cellular density in the nucleus accumbens) during childhood (9–10 years old) were associated with body mass index at the one-year follow-up (*n* = 2212) [122]. The authors discussed the results in the context of obesity and possibly unhealthy eating, although the percentage of obesity or presence of unhealthy eating could not be reported [122]. Another study of the same cohort of children evidenced that higher functional resting state connectivity between the nucleus accumbens and the frontoparietal network was predictive of BMI increase over time, although only for the female group [123]. In adolescents, one study in a community sample (*n* = 122) observed that those with BED symptoms (possibly a mixed sample sBED/BED), compared to those without, showed an increased reward-receipt response in the caudate in a reward-guessing task when money was won [95]. In this same study, the activation of both the ventromedial prefrontal cortex and of the caudate during reward receipt correlated positively with binge symptoms severity; in addition, there were no between-group activity differences during the anticipation of reward in any of the selected regions (striatum, medial prefrontal cortex, orbitofrontal cortex, and amygdala) [95].

BED has received more attention in the study of reward-based neural responses, but mostly in adults. A recent systematic review concluded that BED and sBED were characterized by lower resting frontostriatal connectivity, but higher activation of this neural system when anticipating or receiving food (see [75], adult BED studies [124,125,126,127,128,129,130,131,132], with only one including adolescents with BED, a resting-state study [133]). In this review, studies in adults with BED also showed the hyperactivation of the insula during the anticipation of reward, but a lower activation when receiving the reward [75]. Another study in a mixed sample of adults with bulimia nervosa and binge-eating disorder suggested differential reward-receipt responses depending on the stimulus: increased activity in reward-processing regions when receiving food, but no differences in response to monetary reward [134]. Other studies also reported that reward-based responses might vary according to homeostatic state [87]; for example, in preclinical models of BED, the normal decreases in food reward value at the orbitofrontal cortex when satiated [135] are attenuated [87], akin to what is observed in humans in bulimia nervosa [136]. However, to our knowledge, the interaction between homeostatic and reward processes has not been explored in BED or sBED samples.

In summary, and in accordance with the existing literature, the observed behavioural increased sensitivity to reward in sBED and BED is complemented by some evidence of the hyperactivation of hub regions of the reward system, or hyperconnectivity between these and prefrontal regions in sBED and BED. Research is again very scarce in youth BED samples, and studies evaluating brain responses to reward in its different processes (e.g., anticipation, receipt, learning, delay), homeostatic states (i.e., fasting, satiety), and in response to different stimuli (i.e., money, food, others) are lacking.

### 4.3. Beyond Inhibition and Reward

Although we described the different processes that may be implicated in sBED conditions and full BED forms (e.g., inhibition and reward-based processes) independently, they are, however, interrelated, with reciprocal influences of one brain system with another, and partially reliant on overlapping brain systems [77,137]. Indeed, based on the restricted literature on youth and the larger body of literature on adults, one possibility is that BED is better explained by an imbalance between the two systems, rather than independent alterations in either of them. Interestingly, one preclinical study provides support for this idea; the researchers found evidence that changes in the connectivity between prefrontal (i.e., medial prefrontal cortex) and nucleus accumbens might lead either to vulnerability or resilience to an addiction-like behaviour with food intake [138]. In particular, the enhancement of synaptic excitatory transmission in this circuit (both at the dorsolateral prefrontal cortex and nucleus accumbens) prevented such behaviour, while the inhibition of neuronal activity in this pathway (dorsolateral prefrontal cortex, in its projections to the nucleus accumbens) led to compulsive food seeking [138]. This provides specific brain targets of vulnerability to be evaluated in humans.

In turn, the presence of sBED conditions is also believed to challenge the homeostatic regulation of eating behaviour (and thus the balance between hunger and satiety), thereby increasing the risk of food overconsumption and health problems (e.g., obesity, diabetes) [139]. Connections from the basolateral amygdala to the lateral hypothalamus during satiety have been implicated in susceptibility to weight gain both in rodents and humans [140], and in one study in adolescents with excess weight, increased resting-state connectivity between the lateral hypothalamus and midbrain was associated with sBED [141]. In a systematic review in children and adolescents, sBED conditions were associated with alterations in frontostriatal and frontoparietal regions involved in self-regulatory processes, but also in regions involved in satiety signalling and interception [13].

Other studies support the idea that alterations in limbic regions would significantly contribute to the expression of full BED forms. This is congruent with clinical observations that binges take place in response to stress and negative affects [19,91] and with, for example, evidence for greater secretion of stress hormones and enzymes (i.e., salivary cortisol, alpha-amylase) in women with BED compared to healthy controls in response to a social stress-inducing task [142]. The few studies that have assessed the limbic system in full BED forms point toward alterations in the amygdala, the anterior insula, the hippocampus, ventral regions of the anterior cingulate cortex, and ventromedial prefrontal and orbitofrontal cortices [91,143]. For example, a neuroimaging study with a small sample of BED women showed a decreased activation of the hippocampus when exposed to unpleasant (physical, social) stressors [144]. Finally, a recent study evaluated connectivity between different brain systems during the resting state in pre-adolescent children with BED compared to healthy children, finding aberrant connectivity in prefrontal to amygdala and in anterior cingulate cortex to orbitofrontal cortex regions [133]. However, to our knowledge, there is no other information on youth with BED in this respect.

### 4.4. Relevant Considerations

A final point needs to be made regarding obesity. Obesity is estimated to be comorbid in 87% of individuals with BED, over the course of their lives [145]. This condition is a potential confounder in studies in BED; it is associated with gliosis and neuroinflammation in reward brain regions [146], and obesity in adulthood has been associated with similar brain inhibitory processing alterations (e.g., lower prefrontal activity) to obesity with BED [43,118]. Some authors found greater reward response to food cues in obesity (reviewed here [147]), but others, for example in children, failed to observe differences in reward regions’ activity [148]. In addition, body interoceptive awareness is attenuated in overweight and obese individuals [149], and some authors suggest that excess weight in youth could be associated with a decreased insula response to interoceptive signals (i.e., satiation) but increased response toward external food cues [150].

Obesity has frequently been associated with similar neuropsychological and neuroimaging alterations to those in BED, and it is difficult to disentangle common and differing vulnerability and maintaining factors. Control groups in studies need to consider obesity as an important confounder factor. As previously mentioned, the literature is mixed on the independent contribution of obesity and sBED/BED to alterations in brain circuitry (Lavagnino et al. [43], as opposed to other studies [116,117]). Of note, a larger number of studies in obesity exist, when compared to sBED and BED. Due to the high comorbidity between conditions, it is likely that most studies in obesity include a significant number of subjects with sBED/BED; however, such clinical characterization is frequently lacking.

## 5. Ultra-Processed Food and Drinks and BED?

According to the NOVA classification, one of the most commonly used definitions of ultra-processed foods (UPFs) [151] is ingredient formulations that result from series of industrial processes [152] and that are characterized by no or relatively small amounts of minimally processed foods that conserve their nutritional properties. In general, they have low nutrient densities, and they are poor in protein, dietary fibre, and micronutrients. At the same time, they have a high energy density, high contents of saturated and trans fatty acids, added sugars, and salt [151]. Moreover, UPFs have a high content of additives (i.e., sweeteners, colorants, emulsifiers) intended to intensify their sensory qualities, palatability, and attractiveness [153]. They may also contain chemicals acquired through contact materials, such as sophisticated packaging (e.g., bisphenol), and neo-formed contaminants generated during food processing (e.g., acrylamide, acrolein) [154]. UPFs are engineered to be highly rewarding and they are easily accessible, inexpensive, heavily marketed, and habit forming [155]. These characteristics make UPFs different from processed foods, which are identified by the NOVA classification as being made by adding culinary ingredients (e.g., sugar, oil, salt) to simple unprocessed or minimally processed natural foods. In addition, while these foods may contain additives to preserve the original food properties or resist microbial contamination, they do not aim to imitate the sensory qualities of natural foods. Finally, several industrial processes with no domestic equivalents are used in the manufacture of UPF products (e.g., extrusion and moulding, and pre-processing for frying).

UPF consumption is associated with negative health outcomes among children and adolescents, including cardiometabolic risk, asthma [156], and obesity [157,158]. Also, growing evidence in both animals and humans suggests that highly processed foods may trigger addictive processes that drive compulsive patterns of intake. A study by Ayton and colleagues [51] was the first to objectively show that patients with a BED diagnosis, as well as bulimia nervosa, consumed approximately 70% UPF, and that foods consumed in a binge pattern were 100% UPF. This was then substantiated by a second study with a large sample of participants [52]. In adolescents from the general population, UPF consumption has also been associated with sBED conditions, including food addiction [159,160,161], but in addition to internalizing problems [162], depressive symptoms [163], and anxiety-induced disturbances [164]. However, despite these associations, the potential effect of the consumption of UPFs on the brain systems implicated in BED and sBED remains to be understood. This is worrying considering that, in some countries, children are highly exposed to unhealthy foods from two years of age [53], a sensitive period because of the unbalanced neurodevelopment between subcortical and prefrontal brain systems [165].

The first evidence that the consumption of UPFs may be associated with changes in the brain systems underlying sBED and BED came from studies showing that the viewing or anticipation of unhealthy foods in children changes the activation in brain regions implicated in reward and cognitive processes (e.g., the orbitofrontal cortex, and inferior frontal gyrus) [166,167]. These results are congruent with alterations in these brain systems in BED and sBED sample groups [26,91,95,107,133]. In addition, it is also of interest to consider that dietary exposure to high levels of foods rich in saturated fats, added sugar, and salt shifts preference to foods with a higher concentration of these substances [168,169]. The reshaping of the gustatory systems induced by these substances, a mechanism known as chemosensory plasticity, may also affect the processing of taste, and reward processes through interactions with the brain [168]. However, these studies provide generic evidence of the effect of UPF. More compelling evidence comes from the few studies that have explored the direct effect of UPF consumption. The first clinical trial showed that a UPF diet increases fasting glucose, insulin levels, and the hunger hormone ghrelin [170]. Also, a recent study reported prenatal UPF consumption to be negatively associated with verbal functioning, including verbal expression and concept reasoning, in early childhood (4–5 years of age) [171], skills that can predict emotional regulation abilities in early adolescence [172]. Of note, albeit in an adult sample, a recently published study showed the consumption of UPF to be positively associated with depressive symptoms but negatively associated with the grey matter volume within the frontolimbic brain circuits, which in those with obesity also encompassed reward-related brain networks (i.e., the ventral striatum) [173]. These preliminary studies indicate that the consumption of UPFs may interact with emotional processes, as previously suggested in the context of the consumption of unhealthy foods in BED [7,19]. However, to our knowledge, no previous studies have investigated this in pediatric samples.

Regarding the effects of specific components and features of UPF, the content of low-/no-calorie sweeteners (LNCSs) [174,175,176], their organoleptic properties (i.e., taste, texture) [177], and design (e.g., ready to consume) [153] have been associated with reduced satiety and overeating [51]. Regarding LNCSs, neuroimaging studies have provided evidence that the sweet taste in the absence of nutritive carbohydrates may not lead to changes in the functioning of the hypothalamus [178,179] and brain regions of the ventral system (i.e., the nucleus accumbens, and the insula) [180]. Another study found that those subjects that lacked activation of the insula following a non-nutritive sweetened drink also showed higher total energy intake in a subsequent libitum buffet [181], and a recent review showed that subcortical limbic brain regions are among the most commonly reported in neuroimaging studies evaluating the processing of sugars and LNCSs. In addition, the soft texture that characterizes some UPFs makes them easier to chew and swallow, with lower satiation, increased eating rate, and higher overall food intake [177,182]. UPFs are designed to be eaten fast, and it is well known that foods that can be ingested rapidly increase subjective appetite and food intake [183], as well as the risk of overconsumption [184].

Finally, some preliminary studies have reported that the adverse effects of UPF additives on gut health (see a review in [92]) may affect eating behaviours through induced alterations in brain neurotransmission. In this line, a study showed that 6 months of consumption of the artificial sweetener sucralose in drinking water in mice altered host microbiota and related metabolites, including those belonging to the serotonin(5-HT)-precursor tryptophan [185]. Inflammation and oxidative stress associated with a high content of additives [186,187,188], trans fats [189,190,191], and advanced glycation end-products can also alter neurotransmission in the ventral and dorsal systems [192]. This is of concern if we consider that a proinflammatory immune profile has been reported by some studies in people with BED and sBED forms [193]. In addition, higher doses or exposure to certain nanoparticles (like those contained in food additives) in mice are associated with induced impairment in DA and 5-HT neurotransmitters [194,195], cytotoxicity in glial cells and hippocampal neurons [196], and hippocampal neuroinflammation [197]. Their accumulation has been demonstrated in the hippocampus, hypothalamus, and cerebral cortex [186,198]. However, further studies should explore whether food-grade nanoparticles have similar effects.

## 6. Conclusions: Future

**Figure 1:**
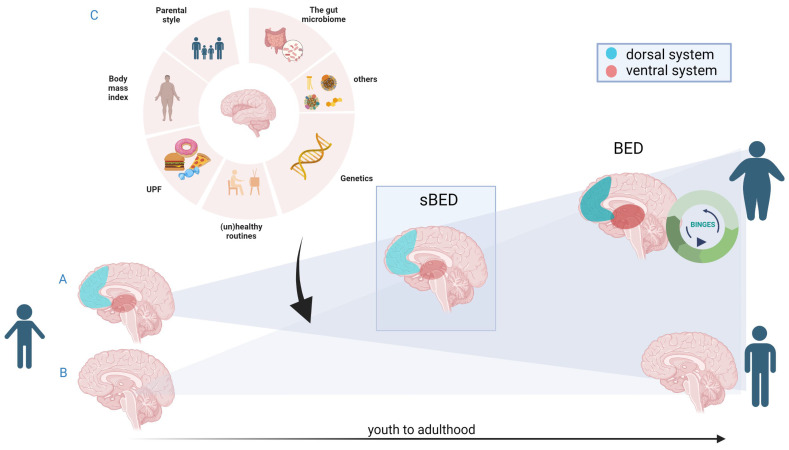
Summary of theoretical approaches to BED emergence in the context of neurodevelopment. From either a vulnerable (**A**) or healthy (**B**) starting point, and subject to external factors (**C**), a sBED condition may or may not develop. From sBED, one group of subjects will develop BED, while others will not.

Despite the scarce existing literature on sBED and BED in youths, the present review established a parallelism in the impaired brain systems between these conditions (see Figure 1). In particular, some evidence points towards a lack of emotional regulatory mechanisms in BED and sBED, mostly involving reward-based processing and inhibitory mechanisms involved in self-regulation, although studies may be biased by the neurocognitive test or MRI task selected. Specifically, evidence of deficits in inhibitory control regions has been found in youth with sBED and prior to sBED development, suggesting them as potential early markers of sBED and possibly of BED. Additionally, studies have indicated that hyperactivation in these regions among youth with sBED may represent potential early brain compensatory mechanisms. In the case of BED, findings come from adult studies, which indicate hypoactivation in inhibitory control circuits. In contrast, hyperactivation of hub regions of the reward system seems to characterize both sBED and BED, as indicated by data from adult studies and some studies involving youth. Of note, obesity is an important confounding factor in most of these findings, but it is rarely taken into account. However, because the diagnosis of BED is mostly based on behaviour (except for the loss of control item), it is possible that different mechanisms involving an imbalance of inhibition and reward-based systems could lead to a similar phenotypic presentation [199]. This is, however, speculative at this point. It is likely that a more complex interplay between brain systems is present, and other systems, such as those involved in interoceptive processes and emotional identification, as well as emotional response, are gaining evidence. More complex analysis regarding brain dynamics will probably help improve our understanding of such altered patterns in BED, particularly during changing neurodevelopmental periods.

A critical evaluation must be conducted regarding the potential association between UPF consumption and the development of primary emotional regulation strategies, and eating behaviours [98]. Excessive UPF exposure prior to adolescence may induce changes in the frontolimbic brain circuits, as well as difficulties in emotional regulation processes at adolescent stages. The risks should not be minimized regarding sBED/BED, and given that UPF consumption is a modifiable factor, preventive and more strictly holistic strategies should be enforced. This is even more important, considering that behavioural interventions remain modest for BED [200]. Information about the potential interaction between UPF consumption and age-related development vulnerability windows, as well as the ‘toxic quantity’ of UPF that each subject might tolerate, should be examined in future studies.

With all the information reviewed in the present manuscript, it becomes apparent that more clarity must be achieved in respect to groups of subjects with sBED conditions that might be more vulnerable to the development of BED. More information about longitudinal trajectories, and the risks and protective factors of BED development is needed. In this regard, neuroimaging biomarkers might prove more valuable in clinical practice for prognostic, rather than diagnostic purposes [201], and they might open up opportunities to develop target-directed treatments (e.g., cognitive rehabilitation, neuromodulation strategies). Finally, one other important question will need to be addressed in future studies: whether sBED is part of a dimensional continuum with BED or, rather, sBEDs are non-pathological traits present in the general population that confer a risk for BED only in vulnerable subjects.

## Data Availability

Not applicable.

## References

[1] American Psychiatric Association (2013). Diagnostic and Statistical Manual of Mental Disorders.

[2] Santomauro D.F., Melen S., Mitchison D., Vos T., Whiteford H., Ferrari A.J. (2021). The hidden burden of eating disorders: An extension of estimates from the Global Burden of Disease Study 2019. Lancet Psychiatry.

[3] Smink F.R.E.E., Van Hoeken D., Oldehinkel A.J., Hoek H.W. (2014). Prevalence and severity of DSM-5 eating disorders in a community cohort of adolescents. Int. J. Eat. Disord..

[4] Kjeldbjerg M.L., Clausen L. (2021). Prevalence of binge-eating disorder among children and adolescents: A systematic review and meta-analysis. Eur. Child Adolesc. Psychiatry.

[5] Tanofsky-Kraff M., Marcus M.D., Yanovski S.Z., Yanovski J.A. (2008). Loss of control eating disorder in children age 12 years and younger: Proposed research criteria. Eat. Behav..

[6] Decaluwé V., Braet C. (2003). Prevalence of binge-eating disorder in obese children and adolescents seeking weight-loss treatment. Int. J. Obes..

[7] Marzilli E., Cerniglia L., Cimino S. (2018). A narrative review of binge eating disorder in adolescence: Prevalence, impact, and psychological treatment strategies. Adolesc. Health Med. Ther..

[8] Ágh T., Kovács G., Supina D., Pawaskar M., Herman B.K., Vokó Z., Sheehan D.V. (2016). A systematic review of the health-related quality of life and economic burdens of anorexia nervosa, bulimia nervosa, and binge eating disorder. Eat. Weight Disord..

[9] World Health Organization (WHO) Obesity and Overweight. https://www.who.int/news-room/fact-sheets/detail/obesity-and-overweight.

[10] Guerdjikova A.I., Mori N., Casuto L.S., McElroy S.L. (2019). Update on Binge Eating Disorder. Med. Clin. N. Am..

[11] Kessler R.C., Berglund P.A., Chiu W.T., Deitz A.C., Hudson J.I., Shahly V., Aguilar-Gaxiola S., Alonso J., Angermeyer M.C., Benjet C. (2013). The prevalence and correlates of binge eating disorder in the World Health Organization World Mental Health Surveys. Biol. Psychiatry.

[12] Neumark-Sztainer D., Wall M., Guo J., Story M., Haines J., Eisenberg M. (2006). Obesity, disordered eating, and eating disorders in a longitudinal study of adolescents: How do dieters fare 5 years later?. J. Am. Diet. Assoc..

[13] Smith K.E., Luo S., Mason T.B. (2021). A systematic review of neural correlates of dysregulated eating associated with obesity risk in youth. Neurosci. Biobehav. Rev..

[14] Vainik U., Neseliler S., Konstabel K., Fellows L.K., Dagher A. (2015). Eating traits questionnaires as a continuum of a single concept. Uncontrolled eating. Appetite.

[15] Hebebrand J., Gearhardt A.N. (2021). The concept of “food addiction” helps inform the understanding of overeating and obesity: NO. Am. J. Clin. Nutr..

[16] Eldredge K.L., Agras W.S. (1996). Weight and shape overconcern and emotional eating in binge eating disorder. Int. J. Eat. Disord..

[17] Nakamura Y., Koike S. (2021). Association of Disinhibited Eating and Trait of Impulsivity with Insula and Amygdala Responses to Palatable Liquid Consumption. Front. Syst. Neurosci..

[18] Tanofsky-Kraff M., Shomaker L.B., Olsen C., Roza C.A., Wolkoff L.E., Columbo K.M., Raciti G., Zocca J.M., Wilfley D.E., Yanovski S.Z. (2011). A Prospective study of pediatric loss of control eating and psychological outcomes. J. Abnorm. Psychol..

[19] Vannucci A., Nelson E.E., Bongiorno D.M., Pine D.S., Yanovski J.A., Tanofsky-Kraff M. (2015). Behavioral and neurodevelopmental precursors to binge-type eating disorders: Support for the role of negative valence systems. Psychol. Med..

[20] Davis C., Carter J.C. (2009). Compulsive overeating as an addiction disorder. A review of theory and evidence. Appetite.

[21] Von Deneen K.M., Liu Y. (2011). Obesity as an addiction: Why do the obese eat more?. Maturitas.

[22] Hauck C., Cook B., Ellrott T. (2020). Food addiction, eating addiction and eating disorders. Proc. Nutr. Soc..

[23] Gearhardt A.N., Corbin W.R., Brownell K.D. (2009). Preliminary validation of the Yale Food Addiction Scale. Appetite.

[24] Imperatori C., Fabbricatore M., Vumbaca V., Innamorati M., Contardi A., Farina B. (2016). Food Addiction: Definition, measurement and prevalence in healthy subjects and in patients with eating disorders. Riv. Psichiatr..

[25] Cebolla A., Perpiñá C., Lurbe E., Alvarez-Pitti J., Botella C. (2012). Prevalencia del trastorno por atracón en una muestra clínica de obesos. An. Pediatría.

[26] Shapiro J.R., Woolson S.L., Hamer R.M., Kalarchian M.A., Marcus M.D., Bulik C.M. (2007). Evaluating binge eating disorder in children: Development of the children’s binge eating disorder scale (C-BEDS). Int. J. Eat. Disord..

[27] Marcus M.D., Kalarchian M.A. (2003). Binge eating in children and adolescents. Int. J. Eat. Disord..

[28] Chamay-Weber C., Combescure C., Lanza L., Carrard I., Haller D.M. (2017). Screening Obese Adolescents for Binge Eating Disorder in Primary Care: The Adolescent Binge Eating Scale. J. Pediatr..

[29] Balantekin K.N., Birch L.L., Savage J.S. (2017). Eating in the absence of hunger during childhood predicts self-reported binge eating in adolescence. Eat. Behav..

[30] Herle M., Stavola B., De Hübel C., Abdulkadir M., Ferreira D.S., Loos R.J.F., Bryant-Waugh R., Bulik C.M., Micali N. (2020). A longitudinal study of eating behaviours in childhood and later eating disorder behaviours and diagnoses. Br. J. Psychiatry.

[31] Ashcroft J., Semmler C., Carnell S., Van Jaarsveld C., Wardle J. (2008). Continuity and stability of eating behaviour traits in children. Eur. J. Clin. Nutr..

[32] Brewerton T.D., Rance S.J., Dansky B.S., O’Neil P.M., Kilpatrick D.G. (2014). A comparison of women with child-adolescent versus adult onset binge eating: Results from the National Women’s Study. Int. J. Eat. Disord..

[33] Stice E., Nathan Marti C., Rohde P. (2013). Prevalence, incidence, impairment, and course of the proposed DSM-5 eating disorder diagnoses in an 8-year prospective community study of young women. J. Abnorm. Psychol..

[34] Brockmeyer T., Skunde M., Wu M., Bresslein E., Rudofsky G., Herzog W., Friederich H.C. (2014). Difficulties in emotion regulation across the spectrum of eating disorders. Compr. Psychiatry.

[35] Favieri F., Marini A., Casagrande M. (2021). Emotional regulation and overeating behaviors in children and adolescents: A systematic review. Behav. Sci..

[36] Mischel W., Shoda Y., Rodriguez M.L. (1989). Delay of Gratification in Children. Science..

[37] Stein R.I., Kenardy J., Wiseman C.V., Dounchis J.Z., Arnow B.A., Wilfley D.E. (2007). What’s driving the binge in binge eating disorder?: A prospective examination of precursors and consequences. Int. J. Eat. Disord..

[38] Penzenstadler L., Soares C., Karila L., Khazaal Y. (2019). Systematic Review of Food Addiction as Measured with the Yale Food Addiction Scale: Implications for the Food Addiction Construct. Curr. Neuropharmacol..

[39] Bleck J.R., DeBate R.D., Olivardia R. (2015). The Comorbidity of ADHD and Eating Disorders in a Nationally Representative Sample. J. Behav. Health Serv. Res..

[40] Goldschmidt A.B., Lavender J.M., Hipwell A.E., Stepp S.D., Keenan K. (2017). Emotion Regulation and Loss of Control Eating in Community-Based Adolescents. J. Abnorm. Child Psychol..

[41] Levin R.L., Rawana J.S. (2016). Attention-deficit/hyperactivity disorder and eating disorders across the lifespan: A systematic review of the literature. Clin. Psychol. Rev..

[42] Kalarchian M.A., Marcus M.D. (2012). Psychiatric comorbidity of childhood obesity. Int. Rev. Psychiatry.

[43] Lavagnino L., Arnone D., Cao B., Soares J.C., Selvaraj S. (2016). Inhibitory control in obesity and binge eating disorder: A systematic review and meta-analysis of neurocognitive and neuroimaging studies. Neurosci. Biobehav. Rev..

[44] Stice E., Davis K., Miller N.P., Marti C.N. (2008). Fasting Increases Risk for Onset of Binge Eating and Bulimic Pathology: A 5-Year Prospective Study. J. Abnorm. Psychol..

[45] Da Luz F.Q., Hay P., Gibson A.A., Touyz S.W., Swinbourne J.M., Roekenes J.A., Sainsbury A. (2015). Does severe dietary energy restriction increase binge eating in overweight or obese individuals? A systematic review. Obes. Rev..

[46] Elran-Barak R., Sztainer M., Goldschmidt A.B., Crow S.J., Peterson C.B., Hill L.L., Crosby R.D., Powers P., Mitchell J.E., Le Grange D. (2015). Dietary Restriction Behaviors and Binge Eating in Anorexia Nervosa, Bulimia Nervosa and Binge Eating Disorder: Trans-diagnostic Examination of the Restraint Model. Eat. Behav..

[47] Racine S.E., Burt S.A., Iacono W.G., McGue M., Klump K.L. (2011). Dietary Restraint Moderates Genetic Risk for Binge Eating. J. Abnorm. Psychol..

[48] Conceição E.M., Moreira C.S., de Lourdes M., Ramalho S., Vaz A.R. (2022). Exploring Correlates of Loss of Control Eating in a Nonclinical Sample. Front. Psychol..

[49] Fairburn C.G., Barnes & Noble (2013). Overcoming Binge Eating: The Proven Program to Learn Why You Binge and How You Can Stop.

[50] Ricciardelli L.A., McCabe M.P. (2001). Dietary restraint and negative affect as mediators of body dissatisfaction and bulimic behavior in adolescent girls and boys. Behav. Res. Ther..

[51] Ayton A., Ibrahim A., Dugan J., Galvin E., Wright O.W. (2021). Ultra-processed foods and binge eating: A retrospective observational study. Nutrition.

[52] Figueiredo N., Kose J., Srour B., Julia C., Kesse-Guyot E., Péneau S., Allès B., Paz Graniel I., Chazelas E., Deschasaux-Tanguy M. (2022). Ultra-processed food intake and eating disorders: Cross-sectional associations among French adults. J. Behav. Addict..

[53] Wang L., Martínez Steele E., Du M., Pomeranz J.L., O’Connor L.E., Herrick K.A., Luo H., Zhang X., Mozaffarian D., Zhang F.F. (2021). Trends in Consumption of Ultraprocessed Foods Among US Youths Aged 2–19 Years, 1999–2018. JAMA.

[54] Marino M., Puppo F., Del Bo’ C., Vinelli V., Riso P., Porrini M., Martini D. (2021). A systematic review of worldwide consumption of ultra-processed foods: Findings and criticisms. Nutrients.

[55] Latasa P., Louzada M.L.D.C., Martinez Steele E., Monteiro C.A. (2018). Added sugars and ultra-processed foods in Spanish households (1990–2010). Eur. J. Clin. Nutr..

[56] Pagliai G., Dinu M., Madarena M.P., Bonaccio M., Iacoviello L., Sofi F. (2021). Consumption of ultra-processed foods and health status: A systematic review and meta-analysis. Br. J. Nutr..

[57] Mitchison D., Touyz S., González-Chica D.A., Stocks N., Hay P. (2017). How abnormal is binge eating? 18-Year time trends in population prevalence and burden. Acta Psychiatr. Scand..

[58] Bulik C.M., Sullivan P.F., Kendler K.S. (2003). Genetic and environmental contributions to obesity and binge eating. Int. J. Eat. Disord..

[59] Frank G.K.W. (2016). The Perfect Storm—A Bio-Psycho-Social Risk Model for Developing and Maintaining Eating Disorders. Front. Behav. Neurosci..

[60] Costa A., Oliveira A. (2023). Parental Feeding Practices and Children’s Eating Behaviours: An Overview of Their Complex Relationship. Healthcare.

[61] Paroche M.M., Caton S.J., Vereijken C.M.J.L., Weenen H., Houston-Price C. (2017). How Infants and Young Children Learn about Food: A Systematic Review. Front. Psychol..

[62] Vaughn A.E., Ward D.S., Fisher J.O., Faith M.S., Hughes S.O., Kremers S.P.J., Musher-Eizenman D.R., O’Connor T.M., Patrick H., Power T.G. (2016). Fundamental constructs in food parenting practices: A content map to guide future research. Nutr. Rev..

[63] Giel K.E., Bulik C.M., Fernandez-Aranda F., Hay P., Keski-Rahkonen A., Schag K., Schmidt U., Zipfel S. (2022). Binge eating disorder. Nat. Rev. Dis. Prim..

[64] Kaye W.H., Fudge J.L., Paulus M. (2009). New insights into symptoms and neurocircuit function of anorexia nervosa. Nat. Rev. Neurosci..

[65] Tepper B.J., Barbarossa I.T. (2020). Taste, Nutrition, and Health. Nutrients.

[66] Small D.M., Prescott J. (2005). Odor/taste integration and the perception of flavor. Exp. Brain Res..

[67] Rolls E.T. (2012). Taste, olfactory and food texture reward processing in the brain and the control of appetite. Proc. Nutr. Soc..

[68] Shepherd G.M. (2006). Smell images and the flavour system in the human brain. Nature.

[69] Small D.M. (2010). Taste representation in the human insula. Brain Struct. Funct..

[70] Chikazoe J., Lee D.H., Kriegeskorte N., Anderson A.K. (2019). Distinct representations of basic taste qualities in human gustatory cortex. Nat. Commun..

[71] Seabrook L.T., Borgland S.L. (2020). The orbitofrontal cortex, food intake and obesity. J. Psychiatry Neurosci..

[72] Dagher A., Neseliler S., Han J.E. (2017). Appetite as Motivated Choice: Hormonal and Environmental Influences. Decis. Neurosci. Integr. Perspect..

[73] Kaye W.H., Wagner A., Fudge J.L., Paulus M. (2011). Neurocircuity of eating disorders. Curr. Top. Behav. Neurosci..

[74] Shapiro A.L.B., Johnson S.L., Sutton B., Legget K.T., Dabelea D., Tregellas J.R. (2019). Eating in the absence of hunger in young children is related to brain reward network hyperactivity and reduced functional connectivity in executive control networks. Pediatr. Obes..

[75] Leenaerts N., Jongen D., Ceccarini J., Van Oudenhove L., Vrieze E. (2022). The neurobiological reward system and binge eating: A critical systematic review of neuroimaging studies. Int. J. Eat. Disord..

[76] Monosov I.E. (2017). Anterior cingulate is a source of valence-specific information about value and uncertainty. Nat. Commun..

[77] Weafer J., Crane N.A., Gorka S.M., Phan K.L., de Wit H. (2019). Neural Correlates of Inhibition and Reward are Negatively Associated. Neuroimage.

[78] Kelley A.E., Baldo B.A., Pratt W.E., Will M.J. (2005). Corticostriatal-hypothalamic circuitry and food motivation: Integration of energy, action and reward. Physiol. Behav..

[79] Berridge K.C. (1996). Food reward: Brain substrates of wanting and liking. Neurosci. Biobehav. Rev..

[80] O’Doherty J.P., Deichmann R., Critchley H.D., Dolan R.J. (2002). Neural responses during anticipation of a primary taste reward. Neuron.

[81] Small D.M., Jones-Gotman M., Dagher A. (2003). Feeding-induced dopamine release in dorsal striatum correlates with meal pleasantness ratings in healthy human volunteers. Neuroimage.

[82] Craig A.D. (2009). How do you feel--now? The anterior insula and human awareness. Nat. Rev. Neurosci..

[83] Craig A.D. (2003). Interoception: The sense of the physiological condition of the body. Curr. Opin. Neurobiol..

[84] Li J., An R., Zhang Y., Li X., Wang S. (2012). Correlations of macronutrient-induced functional magnetic resonance imaging signal changes in human brain and gut hormone responses. Am. J. Clin. Nutr..

[85] Simmons W.K., Rapuano K.M., Kallman S.J., Ingeholm J.E., Miller B., Gotts S.J., Avery J.A., Hall K.D., Martin A. (2013). Category-specific integration of homeostatic signals in caudal but not rostral human insula. Nat. Neurosci..

[86] Frank S., Kullmann S., Veit R. (2013). Food related processes in the insular cortex. Front. Hum. Neurosci..

[87] Romei A., Voigt K., Verdejo-Garcia A. (2020). A Perspective on Candidate Neural Underpinnings of Binge Eating Disorder: Reward and Homeostatic Systems. Curr. Pharm. Des..

[88] Wang G.J., Volkow N.D., Telang F., Jayne M., Ma J., Rao M., Zhu W., Wong C.T., Pappas N.R., Geliebter A. (2004). Exposure to appetitive food stimuli markedly activates the human brain. Neuroimage.

[89] Pelchat M.L., Johnson A., Chan R., Valdez J., Ragland J.D. (2004). Images of desire: Food-craving activation during fMRI. Neuroimage.

[90] Contreras-Rodríguez O., Cano M., Vilar-López R., Rio-Valle J.S., Verdejo-Román J., Navas J.F., Martín-Pérez C., Fernández-Aranda F., Menchón J.M., Soriano-Mas C. (2019). Visceral adiposity and insular networks: Associations with food craving. Int. J. Obes..

[91] Wonderlich J.A., Bershad M., Steinglass J.E. (2021). Exploring Neural Mechanisms Related to Cognitive Control, Reward, and Affect in Eating Disorders: A Narrative Review of FMRI Studies. Neuropsychiatr. Dis. Treat..

[92] Contreras-Rodriguez O., Escorihuela R.M. (2022). Dissecting ultra—Processed foods and drinks: Do they have a potential to impact the brain?. Rev. Endocr. Metab. Disord..

[93] Berthoud H.R. (2004). Neural control of appetite: Cross-talk between homeostatic and non-homeostatic systems. Appetite.

[94] Berthoud H.R. (2006). Homeostatic and non-homeostatic pathways involved in the control of food intake and energy balance. Obesity.

[95] Bodell L.P., Wildes J.E., Goldschmidt A.B., Lepage R., Keenan K.E., Guyer A.E., Hipwell A.E., Stepp S.D., Forbes E.E. (2018). Associations between Neural Reward Processing and Binge Eating among Adolescent Girls. J. Adolesc. Health.

[96] Gross J.J. (2015). The Extended Process Model of Emotion Regulation: Elaborations, Applications, and Future Directions. Psychol. Inq..

[97] Somerville L., Casey B. (2010). Developmental neurobiology of cognitive control and motivational systems. Curr. Opin. Neurobiol..

[98] Willner C.J., Hoffmann J.D., Bailey C.S., Harrison A.P., Garcia B., Ng Z.J., Cipriano C., Brackett M.A. (2022). The Development of Cognitive Reappraisal from Early Childhood through Adolescence: A Systematic Review and Methodological Recommendations. Front. Psychol..

[99] Berner L.A., Marsh R. (2014). Frontostriatal circuits and the development of bulimia nervosa. Front. Behav. Neurosci..

[100] Ochsner K.N., Silvers J.A., Buhle J.T. (2012). Review and evolving model of the cognitive control of emotion. Ann. N. Y. Acad. Sci..

[101] McRae K., Gross J.J., Weber J., Robertson E.R., Sokol-Hessner P., Ray R.D., Gabrieli J.D.E., Ochsner K.N. (2012). The development of emotion regulation: An fMRI study of cognitive reappraisal in children, adolescents and young adults. Soc. Cogn. Affect. Neurosci..

[102] Morris A.S., Criss M.M., Silk J.S., Houltberg B.J. (2017). The Impact of Parenting on Emotion Regulation during Childhood and Adolescence. Child Dev. Perspect..

[103] Cimino S., Marzilli E., Tafà M., Cerniglia L. (2020). Emotional-Behavioral Regulation, Temperament and Parent–Child Interactions Are Associated with Dopamine Transporter Allelic Polymorphism in Early Childhood: A Pilot Study. Int. J. Environ. Res. Public Health.

[104] Zalewski M., Lengua L.J., Wilson A.C., Trancik A., Bazinet A. (2011). Emotion Regulation Profiles, Temperament, and Adjustment Problems in Preadolescents. Child Dev..

[105] Barzman D., Geise C., Lin P.-I. (2015). Review of the genetic basis of emotion dysregulation in children and adolescents. World J. Psychiatry.

[106] Zeman J., Cassano M., Perry-Parrish C., Stegall S. (2006). Emotion regulation in children and adolescents. J. Dev. Behav. Pediatr..

[107] Bartholdy S., O’Daly O.G., Campbell I.C., Banaschewski T., Barker G., Bokde A.L.W., Bromberg U., Büchel C., Burke Quinlan E., Desrivieres S. (2019). Neural Correlates of Failed Inhibitory Control as an Early Marker of Disordered Eating in Adolescents. Biol. Psychiatry.

[108] Olsavsky A.K., Shott M.E., Deguzman M.C., Frank G.K.W.W. (2019). Neural correlates of taste reward value across eating disorders. Psychiatry Res. Neuroimaging.

[109] Treasure J., Duarte T.A., Schmidt U., Antunes Duarte T., Schmidt U. (2020). Eating disorders. Lancet.

[110] Hardee J.E., Phaneuf C., Cope L., Zucker R., Gearhardt A., Heitzeg M. (2020). Neural correlates of inhibitory control in youth with symptoms of food addiction. Appetite.

[111] Jarcho J.M., Tanofsky-Kraff M., Nelson E.E., Engel S.G., Vannucci A., Field S.E., Romer A.L., Hannallah L., Brady S.M., Demidowich A.P. (2015). Neural activation during anticipated peer evaluation and laboratory meal intake in overweight girls with and without loss of control eating. Neuroimage.

[112] Goldschmidt A.B., Dickstein D.P., MacNamara A.E., Phan K.L., O’Brien S., Le Grange D., Fisher J.O., Keedy S. (2018). A pilot study of neural correlates of loss of control eating in children with overweight/obesity: Probing intermittent access to food as a means of eliciting disinhibited eating. J. Pediatr. Psychol..

[113] ABCD Study US Department of Health & Human Services (HHS) ABCD Study. https://abcdstudy.org/.

[114] Schumann G., Loth E., Banaschewski T., Barbot A., Barker G., Büchel C., Conrod P.J., Dalley J.W., Flor H., Gallinat J. (2010). The IMAGEN study: Reinforcement-related behaviour in normal brain function and psychopathology. Mol. Psychiatry.

[115] Frank G.K.W., Shott M.E., DeGuzman M.C. (2019). The Neurobiology of Eating Disorders. Child Adolesc. Psychiatr. Clin. N. Am..

[116] Balodis I.M., Molina N.D., Kober H., Worhunsky P.D., White M.A., Sinha R., Grilo C.M., Potenza M.N. (2013). Divergent neural substrates of inhibitory control in binge eating disorder relative to other manifestations of obesity. Obesity.

[117] Hege M.A., Stingl K.T., Kullmann S., Schag K., Giel K.E., Zipfel S., Preissl H. (2015). Attentional impulsivity in binge eating disorder modulates response inhibition performance and frontal brain networks. Int. J. Obes..

[118] Stice E., Burger K. (2019). Neural vulnerability factors for obesity. Clin. Psychol. Rev..

[119] Frank G.K.W. (2013). Altered brain reward circuits in eating disorders: Chicken or egg?. Curr. Psychiatry Rep..

[120] Hartmann A.S., Czaja J., Rief W., Hilbert A. (2010). Personality and psychopathology in children with and without loss of control over eating. Compr. Psychiatry.

[121] Giel K.E., Teufel M., Junne F., Zipfel S., Schag K. (2017). Food-Related Impulsivity in Obesity and Binge Eating Disorder-A Systematic Update of the Evidence. Nutrients.

[122] Rapuano K.M., Laurent J.S., Hagler D.J., Hatton S.N., Thompson W.K., Jernigan T.L., Dale A.M., Casey B.J., Watts R. (2020). Nucleus accumbens cytoarchitecture predicts weight gain in children. Proc. Natl. Acad. Sci. USA.

[123] Assari S., Boyce S., Bazargan M. (2020). Nucleus accumbens functional connectivity with the frontoparietal network predicts subsequent change in body mass index for American children. Brain Sci..

[124] Balodis I.M., Kober H., Worhunsky P.D., White M.A., Stevens M.C., Pearlson G.D., Sinha R., Grilo C.M., Potenza M.N. (2013). Monetary reward processing in obese individuals with and without binge eating disorder. Biol. Psychiatry.

[125] Frank G.K.W., Shott M.E., Stoddard J., Swindle S., Pryor T.L. (2021). Association of Brain Reward Response with Body Mass Index and Ventral Striatal-Hypothalamic Circuitry among Young Women with Eating Disorders. JAMA Psychiatry.

[126] Hartogsveld B., Quaedflieg C.W.E.M., van Ruitenbeek P., Smeets T. (2022). Decreased putamen activation in balancing goal-directed and habitual behavior in binge eating disorder. Psychoneuroendocrinology.

[127] Miranda-Olivos R., Steward T., Martínez-Zalacaín I., Mestre-Bach G., Juaneda-Seguí A., Jiménez-Murcia S., Fernández-Formoso J.A., Vilarrasa N., Veciana de las Heras M., Custal N. (2021). The neural correlates of delay discounting in obesity and binge eating disorder. J. Behav. Addict..

[128] Schienle A., Schäfer A., Hermann A., Vaitl D. (2009). Binge-eating disorder: Reward sensitivity and brain activation to images of food. Biol. Psychiatry.

[129] Reiter A.M.F., Heinze H.J., Schlagenhauf F., Deserno L. (2017). Impaired Flexible Reward-Based Decision-Making in Binge Eating Disorder: Evidence from Computational Modeling and Functional Neuroimaging. Neuropsychopharmacology.

[130] Voon V., Joutsa J., Majuri J., Baek K., Nord C.L., Arponen E., Forsback S., Kaasinen V. (2020). The neurochemical substrates of habitual and goal-directed control. Transl. Psychiatry.

[131] Voon V., Derbyshire K., Rück C., Irvine M.A., Worbe Y., Enander J., Schreiber L.R.N., Gillan C., Fineberg N.A., Sahakian B.J. (2015). Disorders of compulsivity: A common bias towards learning habits. Mol. Psychiatry.

[132] Skandali N., Majuri J., Joutsa J., Baek K., Arponen E., Forsback S., Kaasinen V., Voon V. (2022). The neural substrates of risky rewards and losses in healthy volunteers and patient groups: A PET imaging study. Psychol. Med..

[133] Murray S.B., Alba C., Duval C.J., Nagata J.M., Cabeen R.P., Lee D.J., Toga A.W., Siegel S.J., Jann K. (2022). Aberrant functional connectivity between reward and inhibitory control networks in pre-adolescent binge eating disorder. Psychol. Med..

[134] Simon J.J., Skunde M., Walther S., Bendszus M., Herzog W., Friederich H.C. (2016). Neural signature of food reward processing in bulimic-type eating disorders. Soc. Cogn. Affect. Neurosci..

[135] Critchley H.D., Rolls E.T. (1996). Hunger and satiety modify the responses of olfactory and visual neurons in the primate orbitofrontal cortex. J. Neurophysiol..

[136] Ely A.V., Wierenga C.E., Bischoff-Grethe A., Bailer U.F., Berner L.A., Fudge J.L., Paulus M.P., Kaye W.H. (2017). Response in taste circuitry is not modulated by hunger and satiety in women remitted from bulimia nervosa. J. Abnorm. Psychol..

[137] Goldstein R.Z., Volkow N.D. (2002). Drug addiction and its underlying neurobiological basis: Neuroimaging evidence for the involvement of the frontal cortex. Am. J. Psychiatry.

[138] Domingo-Rodriguez L., Ruiz de Azua I., Dominguez E., Senabre E., Serra I., Kummer S., Navandar M., Baddenhausen S., Hofmann C., Andero R. (2020). A specific prelimbic-nucleus accumbens pathway controls resilience versus vulnerability to food addiction. Nat. Commun..

[139] Berthoud H.R., Morrison C.D., Münzberg H. (2020). The obesity epidemic in the face of homeostatic body weight regulation: What went wrong and how can it be fixed?. Physiol. Behav..

[140] Sun X.X., Kroemer N.B., Veldhuizen M.G., Babbs A.E., De Araujo I.E., Gitelman D.R., Sherwin R.S., Sinha R., Small D.M. (2015). Basolateral Amygdala Response to Food Cues in the Absence of Hunger Is Associated with Weight Gain Susceptibility. J. Neurosci..

[141] Martín-Pérez C., Contreras-Rodríguez O., Vilar-López R., Verdejo-García A. (2019). Hypothalamic Networks in Adolescents with Excess Weight: Stress-Related Connectivity and Associations with Emotional Eating. J. Am. Acad. Child Adolesc. Psychiatry.

[142] Naumann E., Svaldi J., Wyschka T., Heinrichs M., von Dawans B. (2018). Stress-induced body dissatisfaction in women with binge eating disorder. J. Abnorm. Psychol..

[143] Phelps E.A., Lempert K.M., Sokol-Hessner P. (2014). Emotion and Decision Making: Multiple Modulatory Neural Circuits. Annu. Rev. Neurosci..

[144] Lyu Z., Jackson T. (2016). Acute Stressors Reduce Neural Inhibition to Food Cues and Increase Eating among Binge Eating Disorder Symptomatic Women. Front. Behav. Neurosci..

[145] Villarejo C., Fernández-Aranda F., Jiménez-Murcia S., Peñas-Lledó E., Granero R., Penelo E., Tinahones F.J., Sancho C., Vilarrasa N., Montserrat-Gil De Bernabé M. (2012). Lifetime obesity in patients with eating disorders: Increasing prevalence, clinical and personality correlates. Eur. Eat. Disord. Rev..

[146] Rapuano K.M., Zieselman A.L., Kelley W.M., Sargent J.D., Heatherton T.F., Gilbert-Diamond D. (2017). Genetic risk for obesity predicts nucleus accumbens size and responsivity to real-world food cues. Proc. Natl. Acad. Sci. USA.

[147] Tomasi D., Volkow N.D. (2013). Striatocortical pathway dysfunction in addiction and obesity: Differences and similarities. Crit. Rev. Biochem. Mol. Biol..

[148] Boutelle K.N., Wierenga C.E., Bischoff-Grethe A., Melrose A.J., Grenesko-Stevens E., Paulus M.P., Kaye W.H. (2015). Increased brain response to appetitive tastes in the insula and amygdala in obese compared with healthy weight children when sated. Int. J. Obes..

[149] Herbert B.M., Pollatos O. (2014). Attenuated interoceptive sensitivity in overweight and obese individuals. Eat. Behav..

[150] Mata F., Verdejo-Roman J., Soriano-Mas C., Verdejo-Garcia A. (2015). Insula tuning towards external eating versus interoceptive input in adolescents with overweight and obesity. Appetite.

[151] Gupta S., Hawk T., Aggarwal A., Drewnowski A. (2019). Characterizing ultra-processed foods by energy density, nutrient density, and cost. Front. Nutr..

[152] Monteiro C.A., Cannon G., Levy R., Moubarac J.-C. (2016). The Food System. NOVA. The star shines bright. Public Health.

[153] Monteiro C.A., Cannon G., Levy R.B., Moubarac J.C., Louzada M.L.C., Rauber F., Khandpur N., Cediel G., Neri D., Martinez-Steele E. (2019). Ultra-processed foods: What they are and how to identify them. Public Health Nutr..

[154] Martínez Steele E., Khandpur N., da Costa Louzada M.L., Monteiro C.A. (2020). Association between dietary contribution of ultra-processed foods and urinary concentrations of phthalates and bisphenol in a nationally representative sample of the US population aged 6 years and older. PLoS ONE.

[155] Moodie R., Stuckler D., Monteiro C., Sheron N., Neal B., Thamarangsi T., Lincoln P., Casswell S. (2013). Profits and pandemics: Prevention of harmful effects of tobacco, alcohol, and ultra-processed food and drink industries. Lancet.

[156] Elizabeth L., Machado P., Zinöcker M., Baker P., Lawrence M. (2020). Ultra-processed foods and health outcomes: A narrative review. Nutrients.

[157] De Amicis R., Mambrini S.P., Pellizzari M., Foppiani A., Bertoli S., Battezzati A., Leone A. (2022). Ultra-processed foods and obesity and adiposity parameters among children and adolescents: A systematic review. Eur. J. Nutr..

[158] Neri D., Steele E.M., Khandpur N., Cediel G., Zapata M.E., Rauber F., Marrón-Ponce J.A., Machado P., da Costa Louzada M.L., Andrade G.C. (2022). Ultraprocessed food consumption and dietary nutrient profiles associated with obesity: A multicountry study of children and adolescents. Obes. Rev..

[159] Schulte E., Avena N., Gerhardt A. (2015). Which foods may be addictive? The roles of processing, fat content, and glycemic load. PLoS ONE.

[160] Filgueiras A.R., Pires de Almeida V.B., Koch Nogueira P.C., Alvares Domene S.M., Eduardo da Silva C., Sesso R., Sawaya A.L. (2019). Exploring the consumption of ultra-processed foods and its association with food addiction in overweight children. Appetite.

[161] Pursey K.M., Davis C., Burrows T.L. (2017). Nutritional Aspects of Food Addiction. Curr. Addict. Rep..

[162] Faisal-Cury A., Leite M.A., Loureiro Escuder M.M., Levy R.B., Fernanda M., Peres T. (2022). The relationship between ultra-processed food consumption and internalising symptoms among adolescents from São Paulo city, Southeast Brazil. Public Health Nutr..

[163] Werneck A.O., Vancampfort D., Oyeyemi A.L., Stubbs B., Silva D.R. (2020). Joint association of ultra-processed food and sedentary behavior with anxiety-induced sleep disturbance among Brazilian adolescents. J. Affect. Disord..

[164] Werneck A.O., Hoare E., Silva D.R. (2021). Do TV viewing and frequency of ultra-processed food consumption share mediators in relation to adolescent anxiety-induced sleep disturbance?. Public Health Nutr..

[165] Swartz J., Monk C. (2014). The Role of Corticolimbic Circuitry in the Development of Anxiety Disorders in Children and Adolescents. Curr. Top. Behav. Neurosci..

[166] Bruce A.S., Bruce J.M., Black W.R., Lepping R.J., Henry J.M., Cherry J.B.C., Martin L.E., Papa V.B., Davis A.M., Brooks W.M. (2014). Branding and a child’s brain: An fMRI study of neural responses to logos. Soc. Cogn. Affect. Neurosci..

[167] Adise S., Geier C.F., Roberts N.J., White C.N., Keller K.L. (2018). Is brain response to food rewards related to overeating? A test of the reward surfeit model of overeating in children. Appetite.

[168] May C.E., Dus M. (2021). Confection Confusion: Interplay between Diet, Taste, and Nutrition. Trends Endocrinol. Metab..

[169] Liu D., Archer N., Duesing K., Hannan G., Keast R. (2016). Mechanism of fat taste perception: Association with diet and obesity. Prog. Lipid Res..

[170] Hall K.D., Ayuketah A., Brychta R., Cai H., Cassimatis T., Chen K.Y., Chung S.T., Costa E., Courville A., Darcey V. (2020). Ultra-Processed Diets Cause Excess Calorie Intake and Weight Gain: An Inpatient Randomized Controlled Trial of Ad Libitum Food Intake. Cell Metab..

[171] Puig-Vallverdú J., Romaguera D., Fernández-Barrés S., Gignac F., Ibarluzea J., Santa-Maria L., Llop S., Gonzalez S., Vioque J., Riaño-Galán I. (2022). The association between maternal ultra-processed food consumption during pregnancy and child neuropsychological development: A population-based birth cohort study. Clin. Nutr..

[172] Griffiths S., Suksasilp C., Lucas L., Sebastian C.L., Norbury C. (2021). Relationship between early language competence and cognitive emotion regulation in adolescence. R. Soc. Open Sci..

[173] Contreras-Rodriguez O., Rales-Moreno M., Fernández-Barrès S., Cimpean A., Arnoriaga-Rodríguez M., Puig J., Biarnés C., Motger-Albertí A., Cano M., José M.F.-R. (2023). Consumption of ultra-processed foods is associated with depression, mesocorticolimbic volume, and inflammation. J. Affect. Disord..

[174] Yunker A.G., Patel R., Page K.A. (2020). Effects of Non-nutritive Sweeteners on Sweet Taste Processing and Neuroendocrine Regulation of Eating Behavior. Curr. Nutr. Rep..

[175] Yeung A.W.K., Wong N.S.M. (2020). How does our brain process sugars and non-nutritive sweeteners differently: A systematic review on functional magnetic resonance imaging studies. Nutrients.

[176] Pepino M.Y. (2015). Physiology & behavior metabolic effects of non-nutritive sweeteners. Physiol. Behav..

[177] De Graaf C., Kok F.J. (2010). Slow food, fast food and the control of food intake. Nat. Rev. Endocrinol..

[178] Smeets P.A.M., De Graaf C., Stafleu A., Van Osch M.J.P., Van Der Grond J. (2005). Functional magnetic resonance imaging of human hypothalamic responses to sweet taste and calories. Am. J. Clin. Nutr..

[179] Van Opstal A.M., Kaal I., van den Berg-Huysmans A.A., Hoeksma M., Blonk C., Pijl H., Rombouts S.A.R.B., van der Grond J. (2019). Dietary sugars and non-caloric sweeteners elicit different homeostatic and hedonic responses in the brain. Nutrition.

[180] Van Opstal A.M., Hafkemeijer A., van den Berg-Huysmans A.A., Hoeksma M., Mulder T.P.J., Pijl H., Rombouts S.A.R.B., van der Grond J. (2021). Brain activity and connectivity changes in response to nutritive natural sugars, non-nutritive natural sugar replacements and artificial sweeteners. Nutr. Neurosci..

[181] Crézé C., Candal L., Cros J., Knebel J.F., Seyssel K., Stefanoni N., Schneiter P., Murray M.M., Tappy L., Toepel U. (2018). The impact of caloric and non-caloric sweeteners on food intake and brain responses to food: A randomized crossover controlled trial in healthy humans. Nutrients.

[182] Bolhuis D.P., Forde C.G., Cheng Y., Xu H., Martin N., De Graaf C. (2014). Slow food: Sustained impact of harder foods on the reduction in energy intake over the course of the day. PLoS ONE.

[183] Krop E.M., Hetherington M.M., Nekitsing C., Miquel S., Postelnicu L., Sarkar A. (2018). Influence of oral processing on appetite and food intake—A systematic review and meta-analysis. Appetite.

[184] Viskaal-van Dongen M., Kok F.J., de Graaf C. (2011). Eating rate of commonly consumed foods promotes food and energy intake. Appetite.

[185] Bian X., Chi L., Gao B., Tu P., Ru H., Lu K. (2017). Gut microbiome response to sucralose and its potential role in inducing liver inflammation in mice. Front. Physiol..

[186] Medina-Reyes E.I., Rodríguez-Ibarra C., Déciga-Alcaraz A., Díaz-Urbina D., Chirino Y.I., Pedraza-Chaverri J. (2020). Food additives containing nanoparticles induce gastrotoxicity, hepatotoxicity and alterations in animal behavior: The unknown role of oxidative stress. Food Chem. Toxicol..

[187] Laster J., Frame L.A. (2019). Beyond the Calories—Is the Problem in the Processing?. Curr. Treat. Options Gastroenterol..

[188] Edalati S., Bagherzadeh F., Asghari Jafarabadi M., Ebrahimi-Mamaghani M. (2021). Higher ultra-processed food intake is associated with higher DNA damage in healthy adolescents. Br. J. Nutr..

[189] Pase C.S., Metz V.G., Roversi K., Roversi K., Vey L.T., Dias V.T., Schons C.F., de David Antoniazzi C.T., Duarte T., Duarte M. (2021). Trans fat intake during pregnancy or lactation increases anxiety-like behavior and alters proinflammatory cytokines and glucocorticoid receptor levels in the hippocampus of adult offspring. Brain Res. Bull..

[190] Trevizol F., Roversi K., Dias V.T., Roversi K., Pase C.S., Barcelos R.C.S., Benvegnu D.M., Kuhn F.T., Dolci G.S., Ross D.H. (2013). Influence of lifelong dietary fats on the brain fatty acids and amphetamine-induced behavioral responses in adult rat. Prog. Neuro Psychopharmacol. Biol. Psychiatry.

[191] Trevizol F., Roversi K.R., Dias V.T., Roversi K., Barcelos R.C.S., Kuhn F.T., Pase C.S., Golombieski R., Veit J.C., Piccolo J. (2015). Cross-generational trans fat intake facilitates mania-like behavior: Oxidative and molecular markers in brain cortex. Neuroscience.

[192] D’cunha N.M., Sergi D., Lane M.M., Naumovski N., Gamage E., Rajendran A., Kouvari M., Gauci S., Dissanayka T., Marx W. (2022). The Effects of Dietary Advanced Glycation End-Products on Neurocognitive and Mental Disorders. Nutrients.

[193] Butler M.J., Perrini A.A., Eckel L.A. (2021). The role of the gut microbiome, immunity, and neuroinflammation in the pathophysiology of eating disorders. Nutrients.

[194] Heidari Z., Mohammadipour A., Haeri P., Ebrahimzadeh-Bideskan A. (2019). The effect of titanium dioxide nanoparticles on mice midbrain substantia nigra. Iran. J. Basic Med. Sci..

[195] Hu R., Gong X., Duan Y., Li N., Che Y., Cui Y., Zhou M., Liu C., Wang H., Hong F. (2010). Neurotoxicological effects and the impairment of spatial recognition memory in mice caused by exposure to TiO_2_ nanoparticles. Biomaterials.

[196] Sheng L., Ze Y., Wang L., Yu X., Hong J., Zhao X., Ze X., Liu D., Xu B., Zhu Y. (2015). Mechanisms of TiO_2_ nanoparticle-induced neuronal apoptosis in rat primary cultured hippocampal neurons. J. Biomed. Mater. Res..

[197] Ze Y., Sheng L., Zhao X., Hong J., Ze X., Yu X., Pan X., Lin A., Zhao Y., Zhang C. (2014). TiO_2_ nanoparticles induced hippocampal neuroinflammation in mice. PLoS ONE.

[198] Węsierska M., Dziendzikowska K., Gromadzka-Ostrowska J., Dudek J., Polkowska-Motrenko H., Audinot J.N., Gutleb A.C., Lankoff A., Kruszewski M. (2018). Silver ions are responsible for memory impairment induced by oral administration of silver nanoparticles. Toxicol. Lett..

[199] Wierenga C.E., Ely A., Bischoff-Grethe A., Bailer U.F., Simmons A.N., Kaye W.H. (2014). Are Extremes of Consumption in Eating Disorders Related to an Altered Balance between Reward and Inhibition?. Front. Behav. Neurosci..

[200] Rajjo T., Mohammed K., Alsawas M., Ahmed A.T., Farah W., Asi N., Almasri J., Prokop L.J., Murad M.H. (2017). Treatment of Pediatric Obesity: An Umbrella Systematic Review. J. Clin. Endocrinol. Metab..

[201] Fullana M.A., Abramovitch A., Via E., López-Sola C., Goldberg X., Reina N., Fortea L., Solanes A., Buckley M.J., Ramella-Cravaro V. (2020). Diagnostic biomarkers for obsessive-compulsive disorder: A reasonable quest or ignis fatuus?. Neurosci. Biobehav. Rev..

